# Supplementary material for: Explore the possible influence of Sjogren’s syndrome on thyroid cancer: A literature data mining and meta-analysis
Source: PLoS One. 2025 Feb 10;20(2):e0318747. doi: 10.1371/journal.pone.0318747 (PMC11809879; doi:10.1371/journal.pone.0318747)
Supplement: Attachment — Submitted filename: Response to Reviewers.docx [file pone.0318747.s004.docx]

Journal Requirements:

When submitting your revision, we need you to address these additional requirements.

1. Please ensure that your manuscript meets PLOS ONE's style requirements, including those for file naming. The PLOS ONE style templates can be found at

<https://journals.plos.org/plosone/s/file?id=wjVg/PLOSOne_formatting_sample_main_body.pdf>

and

<https://journals.plos.org/plosone/s/file?id=ba62/PLOSOne_formatting_sample_title_authors_affiliations.pdf>

Answer: We check the format of the revised manuscript and made necessary changes according to the PLOS ONE style templates. Please let us know if further changes are needed.

2. Thank you for stating the following financial disclosure:

“This work was partially supported by the Beijing Municipal Administration of Hospitals’ Youth Programme (QMS20210206); Scientific Research Common Program of Beijing Municipal Commission of Education (KM202210025014).”

Please state what role the funders took in the study.  If the funders had no role, please state: "The funders had no role in study design, data collection and analysis, decision to publish, or preparation of the manuscript."

If this statement is not correct you must amend it as needed.

Please include this amended Role of Funder statement in your cover letter; we will change the online submission form on your behalf.

Answer: We have the financial statement revised as follows.

*This work was partially supported by the Beijing Municipal Administration of Hospitals’ Youth Programme (QMS20210206); Scientific Research Common Program of Beijing Municipal Commission of Education (KM202210025014). The funders had no role in study design, data collection and analysis, decision to publish, or preparation of the manuscript.*

Reviewer #1: This study mainly used literature data mining and GEO database joint analysis to explore the common genes/proteins between SS and TC, which has a certain degree of innovation. However, the methods of literature data mining in the study were not specifically specified, which raises doubts about the subsequent results. And this study only used relevant information from literature and databases, without further experimental validation analysis of the excavated genes or validation analysis of other databases. The reliability and authenticity of the final results are questionable. I think it is necessary to conduct further validation analysis, whether it is laboratory related analysis such as PCR, WB, IHC, or selecting other databases for validation, such as TC related TGCA database or SS related GEO database.
Here are some specific questions about the article:

1. In methods and materials, the relevant driving factors extracted from literature data mining, and the specific methods of literature data mining? What literature databases have been mined? What are these documents specifically? It is not explicitly stated in the article. These steps are necessary as they relate to the accuracy of subsequent analysis results and the rigor and rationality of the article.

Answer: Thank you for your suggestion. We fully agree that providing this specific information is essential for the audience to understand the accuracy of the subsequent analysis results, as well as the rigor and rationality of the article. We have added more details in the Methods section under ‘SS-driven molecules influencing TC’ and cited the following text:

*To investigate the potential influence of SS on TC, we performed literature data mining (LDM) to identify SS-driven molecules that also act as upstream regulators of TC. The LDM was carried out using Pathway Studio (version 12.3), which utilizes the natural language processing (NLP) tool MedScan [13]. This process involved mining data from 24 million PubMed abstracts and 3.5 million Elsevier and third-party full-text papers. Each relationship or edge was established based on facts extracted from the literature using NLP technology, supported by at least one reference. Key information extracted included relation type, relation polarity, reference title, PMID/DOI, and the key sentences where the relationship was identified.*

2. How can we determine if PDCD1, NTRK1, LGALS3, CD274, FOXP3, BCL2, CYP1130 HMGB1, TGFB1, CCL2, as well as MIR145, EP300, and FOXP3 are activators/inhibitors of TC? What are the relevant literature? Specific instructions are required.
   Answer: Thank you for the question. The identification of these molecules as activators or inhibitors of TC was determined by a Natural Language Processing (NLP) algorithm. Additionally, the relevant information, including specific sentences from the literature, was extracted for manual validation. To enhance transparency and allow for validation by the audience, we included a Reference table in Supplementary Material S1 Table alongside Figure 1. This table provides detailed information such as the Relation Name, Reference, Title, related Sentence, and supporting references (e.g., Source, PMID, PubYear, and DOI). We have also added a detailed description in the Results section under ‘SS-driven molecules pathway influencing TC’ and cited the following text:

*For details on the relationships/edges presented in Fig 1, please refer to Supplementary Material S1 Table, which includes information on the supporting references, such as the reference title, PMID, DOI, and the relevant sentences where the relationships were identified.*

3. How many screened LEF genes are in each of the 16 TC expression databases? How did they end up getting at these genes in Table 2? Need to list them specifically.
   Answer: Thank you for your question. We screened 273 SS-driven molecules and analyzed their expression changes in TC patients compared to controls using 16 TC expression datasets. Out of these 273 molecules, 245 were identified in at least one of the 16 datasets. Except for CD68 and NEAT1, all other genes were identified in at least 10 of the TC expression datasets. The genes listed in Table 2 were selected through a meta-analysis of these 245 genes. We calculated the log2 fold-change (LFC) and p-value across all datasets to determine significance, with genes selected based on an absolute LFC greater than or equal to 1 and a p-value less than 0.05. Depending on the heterogeneity of the datasets, either a random-effects or fixed-effect model was used.

The detailed results for each of these genes, including the meta-analysis model used, the number of datasets (studies) included, and the results of multiple-linear regression analysis, are provided in Supplementary Material S2 Table. This supplementary material ensures transparency and allows for verification of the screening process.

We added a description in the results section "Meta-analysis results," as follows:

*"For the 273 SS-driven molecules, 245 were identified in at least one of the 16 TC expression datasets. Except for CD68 and NEAT1, all other genes were identified in at least 10 TC expression datasets. Detailed meta-analysis results for each of these genes are provided in Supplementary Material S2 Table, including the meta-analysis model used, the number of datasets (studies) involved, LFC, p-value, and the results of multiple-linear regression analysis."*

4. What is the basis for the statement in line 200 that based on literature data mining, SS activated four of these genes (PLA2G7, TFF3, LCN2 and CLDN1) while inhibiting three of them (LTF, TIMP1 and LGALS1)? Relevant literature is needed to support this result.
   Answer: Thank you for the question. We have provided the relevant literature to support this statement in Supplementary Material S3 Table. This material includes detailed references that substantiate the activation of PLA2G7, TFF3, LCN2, and CLDN1 by SS, as well as the inhibition of LTF, TIMP1, and LGALS1. We have added a description in the relevant Results section, "Role of significant genes in meta-analysis in thyroid cancer," and cited it as follows:

*"… and that SS activates four of them (PLA2G7, TFF3, LCN2, and CLDN1), while inhibiting three of them (LTF, TIMP1, and LGALS1). See Supplementary Material S3 Table for the details of the supporting references."*

5. The serial numbers in the article are confusing, e.g., 1.result appears in line 125, but 3.1 driven molecules pathway influencing TC appears in line 126. The format of the article needs to be systematically checked.

Answer: Thank you for pointing this out. We have systematically reviewed and reformatted the article to ensure consistency and adherence to PLOS ONE's style requirements. All serial numbers and section headings have been corrected to improve clarity and organization.

6. The discussion is merely a description of the results and a stack of literature citations, and should focus on describing the key genes in which SS is associated with TC. For example, it is mentioned in the text that CCL2 is up-regulated by SS and PLA2G7 is significantly changed in TC. PLA2G7 can be positively correlated with TC through CCL2 and HIF1A, and CCL2 has been proved to promote metastasis of thyroid cancer. It would be a better direction to summarize the SS-CCL2-PLA2G7-TC pathway and conduct an in-depth discussion and experimental or database validation of this pathway.

Answer: Thank you for the valuable suggestion. Based on the Literature Data Mining (LDM) and Meta-analysis using TC expression datasets, we identified potential pathways that may explain how SS contributes to the pathological development of TC, as illustrated in Figures 1 and 3. By integrating these pathways, we highlighted three key mechanisms—the SS-PLA2G7-CCL2-TC pathway, the SS-LCN2- LGALS3-TC pathway and the SS-CLDN1-BCL2-TC pathway—and conducted an in-depth discussion on their possible mechanisms. We have incorporated these discussions into the Discussion section, as cited below:

*By integrating the pathways presented in Figures 1 and 3, we identified three potential mechanisms through which SS may contribute to the pathological development of TC. These pathways include the SS-PLA2G7-CCL2-TC pathway, the SS-LCN2- LGALS3-TC pathway and the SS-CLDN1-BCL2-TC pathway.*

*As an autoimmune disorder, SS has been associated with an increased risk of TC, with particular emphasis on the regulation of CCL2 and PLA2G7. CCL2, a chemokine involved in inflammation, and PLA2G7, an enzyme linked to lipid metabolism, may interact to create a pro-inflammatory and pro-tumorigenic microenvironment conducive to TC progression. CCL2 is a key player in both SS and TC, where its association with SS disease activity and inflammation may occur through NF-κB pathway activation [32-34]. In TC, CCL2 promotes cell migration and is predictive of papillary thyroid carcinoma, highlighting its significance in tumor progression [35, 36]. Moreover, the modulation of CCL2 by factors such as Canagliflozin, PFHxA, USP15, and Vitamin D further connects its involvement in TC development, particularly in association with papillary thyroid carcinoma and Hashimoto's thyroiditis [35, 37, 38]. PLA2G7, influenced by SS-related factors like second-hand smoke, contributes to SS-associated inflammation and potentially interacts with CCL2 in COPD [39]. The expression and activity of PLA2G7 are also linked to B-cell lymphoma in SS, suggesting its diagnostic utility in SS-related malignancies [40]. The interplay between CCL2 and PLA2G7 in the context of SS and TC underscores the complex regulatory mechanisms underlying the pathogenesis and progression of these interconnected conditions, necessitating further research to elucidate their precise roles.*

*Moreover, the linkage between SS and an increased risk of TC may also be attributed to the regulation of LGALS3 (Galectin-3) and LCN2. LGALS3, known for promoting tumor growth and metastasis in TC, has shown significant associations with TC susceptibility, particularly in papillary thyroid carcinoma. Specific genetic variants of LGALS3 influence the risk of developing TC, and its role in the invasiveness of BRAF-mutated papillary thyroid carcinoma highlights its potential as a therapeutic target in tumor progression [41, 42]. LCN2, which regulates inflammation and immune response in the tumor microenvironment, is overexpressed in aggressive TC variants and is associated with epithelial-mesenchymal transition, making it a valuable predictive and diagnostic biomarker [43, 44]. The influence of both LGALS3 and LCN2 on TC invasiveness via NF-κB pathways further underscores their therapeutic potential in specific TC subtypes [45, 46]. Additionally, the elevation of LGALS3 in SS stages suggests its role as an alarmin, particularly in SS patients with interstitial lung disease, and underscores the intricate interplay between LGALS3, LCN2, SS, and TC. This association between SS and an increased risk of TC, supported by higher incidence rates of TC in patients with primary SS, emphasizes the need for further research to elucidate the underlying mechanisms [47, 48].*

*In addition, SS upregulates CLDN1 and BCL2 [49, 50], two important genes involved in cell proliferation and apoptosis, which may contribute to an increased risk of TC. CLDN1, a tight junction protein, could disrupt normal cell-cell interactions in the thyroid gland, potentially promoting tumorigenesis [51, 52]. Meanwhile, BCL2, an anti-apoptotic protein, might prevent the programmed cell death of cancerous thyroid cells, contributing to tumorigenesis and therapeutic resistance [53]. Thus, the dysregulation of CLDN1 and BCL2 in the context of SS may contribute to the development and progression of TC by influencing cellular processes related to tumorigenesis and therapeutic resistance.*

7. The description of the conclusion is too broad, and the writing structure is incorrect. Specific research points in the article should be summarized, such as what is the relationship between SS and TC? Which common gene affects the incidence of both? As well as the strengths and weaknesses of the article should be specifically described in the conclusion.

Answer: Thank you for the suggestion. We have revised the Conclusion to incorporate the major findings of the study. The revised Conclusion is now cited as follows.

*Conclusion*

*Our study proposed multiple pathways and highlighted three possible mechanisms—the SS-PLA2G7-CCL2-TC pathway, the SS-LCN2-LGALS3-TC pathway, and the SS-CLDN1-BCL2-TC pathway—that may explain how SS contributes to the pathological development of TC. These findings provide valuable insights into the relationship between primary SS and TC and underscore the need for further investigation to fully understand the precise nature of this relationship and its potential implications for TC diagnosis and treatment.*

We also included a discussion of the strengths and limitations of this study, which are cited as follows.

*In summary, this study has notable strengths and limitations that should be addressed in future research. A key strength is the integration of literature data mining with GEO database analysis, which allowed us to explore the potential genetic and molecular contributions of SS to TC. This approach gathered information from a wide range of studies, potentially uncovering hidden evidence of SS-TC interactions. We identified multiple pathways and highlighted three possible mechanisms—the SS-PLA2G7-CCL2-TC pathway, the SS-LCN2-LGALS3-TC pathway, and the SS-CLDN1-BCL2-TC pathway—that may explain how SS contributes to TC development. However, the study's limitations include its reliance on literature and existing TC expression databases, which lack specific SS disease context. Experimental data from SS patients who develop TC should be collected for further validation of the identified genes and pathways.*

Moreover, based on these changes, we have revised the Abstract, which is now cited as follows.

***Abstract***

***Objectives***

*To explore the potential influence of Sjogren's syndrome (SS) on thyroid cancer (TC).*

***Methods***

*First, literature data mining was conducted to reconstruct functional pathways connecting SS and TC. Subsequently, a meta-analysis was performed using 16 TC case/control expression datasets to investigate the expression changes of genes mediated by SS. Finally, gene set enrichment analysis (GSEA) was conducted on the significant genes identified in the expression meta-analysis to identify potential pathways and functional groups influenced by SS in the pathology of TC.*

***Results***

*Our findings indicate that SS may influence the risk of TC through the regulation of 21 molecules. Specifically, SS appears to increase the risk of TC by activating 14 TC promoters (PDCD1, NTRK1, LGALS3, CD274, FOXP3, BCL2, CYP1A1, HMGB1, TGFB1, CCL2, PLA2G7, TFF3, LCN2, and CLDN1) and suppressing three TC inhibitors (MIR145, MIR30C1, and EP300). Moreover, four molecules (PLA2G7, TFF3, LCN2, and CLDN1) exhibited significant expression changes in TC patients (LFC > 1 or < -1; p < 2.07E-04). The overlapped pathways constructed from both LDM and expression data analysis highlighted three possible mechanisms—the SS-PLA2G7-CCL2-TC pathway, the SS-LCN2-LGALS3-TC pathway, and the SS-CLDN1-BCL2-TC pathway—that may explain how SS contributes to TC development. Enrichment analysis suggests that SS may affect TC prognosis by regulating leukocytes and tolerance induction.*

***Conclusion***

*Our findings provide insight into the link between primary SS and TC and highlight the need for further research to fully understand the nature of this relationship and its implications for TC diagnosis and treatment.*
