# Supplementary material for: Explore the possible influence of Sjogren’s syndrome on thyroid cancer: A literature data mining and meta-analysis
Source: PLoS One. 2025 Feb 10;20(2):e0318747. doi: 10.1371/journal.pone.0318747 (PMC11809879; doi:10.1371/journal.pone.0318747)
Supplement: Attachment — Submitted filename: Response to Reviewers.docx [file pone.0318747.s005.docx]

Reviewer #2: This study presents an investigation into the potential molecular pathways linking Sjogren's syndrome (SS) and thyroid cancer (TC). The authors have integrated literature data mining and RNA expression-based meta-analysis to identify genes influenced by SS that may contribute to the pathological development and progression of TC.

However, the comments from Reviewer #1 on the database validation of the highlighted genes and pathways have not been sufficiently addressed. Since the goal of this study is to investigate genes influenced by SS that may also contribute to the pathological development and progression of TC [Lines 74-75, 243-244, 261, 277-278, etc.], the authors need to further investigate these genes using data from TCGA/PanCancer Atlas.

The TCGA has close to 500 TC samples with gene expression data. The GEPIA 2 portal [Tang et al., 2019; 10.1093/nar/gkz430] has readily-available differential expression analysis of TC samples integrated with healthy controls from GTEx. Furthermore, the genes highlighted in the study should be further investigated. For instance, are there hotspot mutations or high mutation-burden in these SS-related genes? Is the expression of these genes linked with severity and/or survival? This information is also readily accessible through cBioPortal and other portals. This will greatly improve the robustness of the findings in the current work by commenting on prognosis or other characteristics of these SS-linked genes in TC.

Answer: Thanks for the suggestion. We further analyzed the identified SS-driven genes and their contribution to the pathological development and progression of TC using TCGA data through GEPIA2 (http://gepia2.cancer-pku.cn/). The manuscript has been updated with these additional analyses, which significantly enhanced the robustness of our current findings. We cited the corresponding changes in each section as follows.

###### Abstract

*Objectives*

*To explore the potential influence of Sjogren's syndrome (SS) on thyroid cancer (TC).*

*Methods*

*First, a literature data mining (LDM) approach was used to reconstruct functional pathways connecting SS and TC. A meta-analysis was then performed to examine the expression changes of genes mediated by SS using 16 TC case/control expression datasets, with results validated through the TCGA/GTEx dataset. Finally, gene set enrichment analysis (GSEA) and survival analysis using GEPIA2 were conducted on the significant genes.*

*Results*

*Our findings indicate that SS may increase the risk of TC by activating 14 TC promoters (PDCD1, NTRK1, LGALS3, CD274, FOXP3, BCL2, CYP1A1, HMGB1, TGFB1, CCL2, PLA2G7, TFF3, LCN2, and CLDN1) and suppressing three TC inhibitors (MIR145, MIR30C1, and EP300). Four molecules (PLA2G7, TFF3, LCN2, and CLDN1) exhibited significant expression changes in TC patients (LFC > 1 or < -1; p < 2.07E-04), which were confirmed in TCGA/GTEx expression analysis. These results highlight three possible mechanisms—the SS-PLA2G7-CCL2-TC pathway, the SS-LCN2-LGALS3-TC pathway, and the SS-CLDN1-BCL2-TC pathway—that may explain how SS contributes to TC development. Enrichment analysis suggests that SS may affect TC prognosis by regulating leukocytes and tolerance induction. Survival analysis indicates that SS may enhance TC survival through the regulation of the CLDN1 and EGF pathways.*

*Conclusion*

*LDM-based pathway analysis highlighted three genetic pathways through which SS may adversely affect TC progression, while SS may enhance TC survival via the CLDN1 and EGF pathways, highlighting the need for further research.*

**Method**

***Analysis of SS-Driven Genes in Thyroid Carcinoma Using TCGA/GTEx data***

*GEPIA2 is an enhanced web server for large-scale expression profiling and interactive analysis [15]. We performed further analysis of the identified SS-driven genes to investigate their contribution to the pathological development and progression of thyroid carcinoma (TC) using the TCGA/GTEx thyroid carcinoma (THCA) dataset. This dataset includes 512 tumor samples, 59 normal samples from the cancer dataset, and 278 normal samples from the GTEx dataset for comparison. In addition to the meta-analysis using the GEO expression dataset, we further analyzed the expression levels of genes involved in the SS-TC pathway and compared them with the results from the meta-analysis. Furthermore, the influence of these genes on THCA survival was tested and compared with their roles in the pathway, using the log-transformed hazard ratio (HR) to represent their impact on thyroid carcinoma.*

**Results**

*3.1 SS-driven molecules pathway influencing TC*

*Figure 1a illustrates the network relationships between SS and TC at the molecular level. SS is associated with the upregulation of ten genes/proteins that promote TC, including PDCD1, NTRK1, LGALS3, CD274, FOXP3, BCL2, CYP1A1, HMGB1, TGFB1, and CCL2. Additionally, SS negatively regulates three genes/proteins that inhibit TC, namely MIR145, EP300, and FOXP3. This suggests that SS may adversely affect TC progression and development through these 13 genetic pathways. For details on the relationships/edges presented in Fig 1a, please refer to Supporting Material S1 Table 1, which includes information on the supporting references, such as the reference title, PMID, DOI, and the relevant sentences where the relationships were identified.*

*However, it is important to note that the genes in the SS-TC pathway depicted in Figure 1a did not exhibit significant expression changes in either the meta-analysis of GEO TC expression data or the THCA dataset, as shown in Figure 1b. Furthermore, none of these 13 genes demonstrated a significant impact on the overall survival of thyroid carcinoma (p(HR) > 0.05), as presented in Figure 1c. These findings highlight the need for further validation of these genetic pathways.*

*In addition, Fig. 1a shows that SS inhibits a promoter of TC, specifically EGF. Survival analysis revealed that EGF is significantly positively associated with the risk of thyroid carcinoma (p(HR) = 0.03) (Fig. 1c). This finding aligns with the SS-EGF-TC pathway presented in Fig. 1a, suggesting that SS may also play a beneficial role in TC survival, highlighting the complex relationship between SS and TC that warrants further investigation.*

**Fig. 1. The Sjogren's syndrome-driven molecular pathway influencing thyroid cancer.** (a) Literature-based pathway connecting SS and TC; (b) Gene expression of the involved genes using the THCA dataset; (c) Survival map of these genes in thyroid carcinoma. Red indicates that higher expression levels are associated with increased risk (unfavorable prognosis), while blue indicates that higher expression levels are associated with reduced risk (favorable prognosis).

***Role of significant genes in meta-analysis in thyroid cancer***

*It is worth noting that the gene expression analysis of these seven genes using TCGA/GTEx thyroid carcinoma (THCA) data confirms the identification results from the meta-analysis of the GEO dataset, as shown in Fig. 3b. Although PLA2G7 and LGALS1 did not reach statistical significance, all these genes exhibited the same direction of variation (increase or decrease) in both the GEO dataset and the THCA dataset.*

*Survival analysis revealed that only CLDN1 is significantly negatively associated with the risk of thyroid carcinoma (p(HR) = 0.031) (Fig. 3c). Additionally, SS has been implicated in promoting CLDN1 (Fig. 3a), suggesting that SS may play a beneficial role in TC survival through the SS-CLDN1-TC pathway.*

***Fig 3 Genes show significant expression change in meta-analysis and their connection with Sjogren’s syndrome and thyroid cancer*** *(a) Literature-based pathway connecting SS and TC; (b) Gene expression of the involved genes using the THCA dataset; (c) Survival map of these genes in thyroid carcinoma. Red indicates that higher expression levels are associated with increased risk (unfavorable prognosis), while blue indicates that higher expression levels are associated with reduced risk (favorable prognosis).*

**Discussion***:*

*Expression analysis using TCGA/GTEx data confirms the expression changes of SS-driven genes that influence TC. Specifically, among the 14 genes in the first pathway (Figure 1a), no significant changes were observed in TC patients compared to normal controls. However, seven genes showed consistent directional variation (either increased or decreased expression) across both the GEO dataset and the THCA dataset. These findings further validate the robustness of our* *meta-analysis.*

*Furthermore, survival analysis revealed that SS may beneficially influence TC survival through two distinct pathways. First, SS promotes CLDN1, which is significantly negatively associated with the risk of thyroid carcinoma (p(HR) = 0.031), suggesting a potential beneficial role in TC survival through the SS-CLDN1-TC pathway. Second, SS inhibits EGF, a promoter of TC that is significantly positively associated with the risk of thyroid carcinoma (p(HR) = 0.03). This aligns with the SS-EGF-TC pathway, further supporting the notion that SS may have a protective role in TC survival. These findings highlight the complex and multifaceted relationship between SS and TC, warranting further investigation.*

**Conclusion**

*Although LDM-based pathway analysis reveals potential pathways through which SS may adversely influence TC progression and development—such as the SS-PLA2G7-CCL2-TC pathway, the SS-LCN2-LGALS3-TC pathway, and the SS-CLDN1-BCL2-TC pathway—SS may also have a beneficial impact on TC survival through the CLDN1 and EGF pathways. These findings underscore the complex relationship between SS and TC, warranting further investigation.*

**Additional comments are as follows:**
Lines 82-85: Approximately when was the literature data mining performed (month, year)? This will indicate the status of the corpus from PubMed and Elsevier included in the mining.

Answer: Thanks for the suggestion. We have added the date information for the LDM, and cited it as follows.

*The relationship data within the molecular network was extracted from the Elsevier Knowledge graph database (*[*www.pathwaystudio.com*](http://www.pathwaystudio.com)*), which covers the entire PubMed database, Elsevier publications, and third-party literature, updated as of February 2024.*

Line 99: Why were expression datasets limited to arrays? Newer, high-throughput/NGS, technologies have higher sensitivities and should be included in the current study.

Answer: Thank you for your insightful comment. We have addressed this point in the Methods section, where the following explanation has been added:

"*It is worth noting that we focused on RNA expression data obtained through arrays rather than NGS data due to compatibility issues with the Meta-analysis tool used in this study. Additionally, much of the NGS data available in GEO is incomplete, and the data formats that are accessible are largely incompatible with our analysis framework. To overcome this limitation, we conducted further analysis of the SS-driven genes that influence TC using TCGA/GTEx thyroid carcinoma (THCA) data.*"

We appreciate your understanding regarding the limitations of data availability and compatibility in this context.

Line 100: Was the filtering criteria SS vs. healthy or TC vs. healthy?

Answer: Thanks for pointing this out. It should be ‘TC vs. healthy’. We have this corrected.
