# Supplementary material for: Explore the possible influence of Sjogren’s syndrome on thyroid cancer: A literature data mining and meta-analysis
Source: PLoS One. 2025 Feb 10;20(2):e0318747. doi: 10.1371/journal.pone.0318747 (PMC11809879; doi:10.1371/journal.pone.0318747)
Supplement: Attachment — Submitted filename: Response to Reviewers.docx [file pone.0318747.s006.docx]

**Reviewer #1:** Thank you for the opportunity to review this manuscript. After carefully evaluating the revised version, I find that the authors have addressed all the review comments thoroughly and have made significant improvements to the quality and clarity of the manuscript.

I am satisfied with the revisions and recommend the manuscript for acceptance and publication.

Answer: Thanks for the comments.

**Reviewer #2:** Thank you for submitting a revised manuscript and for addressing the major comments. For future, a gene set/signature analysis may also be considered when assessing a group of genes (e.g., 14 genes from Figure 1a) with disease progression/severity/treatment response/etc., as seldom do individual genes show striking correlation with disease/clinical characteristics.

Answer: Thank you for the valuable suggestion. We have incorporated this point into the discussion as part of the future directions for this research. The revised text reads as follows:

*"Furthermore, in addition to the individual evaluation of significant genes, a gene signature analysis considering the group of target genes should be conducted to assess their collective significance in TC and SS, particularly in relation to disease progression, severity, and treatment response."*

**Reviewer #3:** This paper has improved a lot now. I have some minor suggestions.
1) Authors should revise the title and embody the detailed work you have conducted, for example: Explore the possible influence of sjogren's syndrome on thyroid cancer: a literature data mining and meta-analysis.

Answer: Thank you for the insightful suggestion. We agree that incorporating more details into the title enhances its clarity and relevance. Accordingly, we have revised the title to: "Explore the Possible Influence of Sjogren's Syndrome on Thyroid Cancer: A Literature Data Mining and Meta-Analysis."

2) Authors should rearrange and restructure all the figures to make them look more sightly.

Answer: Thank you for the valuable suggestion. We agree that the original figures were overly complex and difficult to interpret. In response, we have restructured the figures, increasing the total number from four to six (Fig. 1 to Fig. 6). Two of the original figures were split to provide better focus on specific topics. The legends and value bars have been rearranged to improve clarity. The updated figures are now more organized and easier to understand. These revised figures have been uploaded along with the revised manuscript, and the figure numbers have been updated accordingly in the main text.
