# Supplementary material for: Explore the possible influence of Sjogren’s syndrome on thyroid cancer: A literature data mining and meta-analysis
Source: PLoS One. 2025 Feb 10;20(2):e0318747. doi: 10.1371/journal.pone.0318747 (PMC11809879; doi:10.1371/journal.pone.0318747)
Supplement: Attachment — Submitted filename: Response to Reviewers.docx [file pone.0318747.s007.docx]

Additional Editor Comments:
- Please redraw the figure 1 and figure 4. The arrows in these figures is not clear. Consider using more solid arrows that are not overlapping. Also please be careful about the names and the shapes. These figures can be more stylish and informative. Please provide the figures according to the journal requirements.

Answer: Figure 1 and Figure 4 have been re-generated using Cytoscape, resulting in a clearer and more visually appealing design. The updated figures adhere to the journal's requirements and have been uploaded as part of the revision. Below are the two revised figures.

**Fig. 1. The Sjogren's syndrome-driven, literature-based molecular pathway influencing thyroid cancer.** Edges in red represent a negative relationship, while those in green represent a positive relationship. A gene in red indicates that SS exerts a detrimental effect on TC through the gene, whereas a gene in green indicates that SS exerts a beneficial effect on TC through the gene.

**Fig 4 Genes show significant expression change in meta-analysis and their connection with Sjogren’s syndrome and thyroid cancer.** Edges in red represent a negative relationship, while those in green represent a positive relationship. A gene in red indicates significantly increased expression in the meta-analysis, whereas a gene in blue indicates significantly decreased expression in the meta-analysis.

- What is the reason for highlighting some items in the Table S1?

Answer: The items in Table S1 were highlighted during the discussion of each gene's information and were inadvertently left in the final version of the Supplementary Table. The highlights have been removed, and the updated Table S1 have been uploaded as part of the revision

- Please double check the citation of Tables throughout the text. I recommend to give more description on the supplementary data.

Answer: We have thoroughly checked the citations of the tables throughout the text and provided additional descriptions of the supplementary data within the main text. The citations have been updated as follows.

*For details on the relationships/edges presented in Fig. 1, please refer to Supporting Material S1 Table, which includes information on the supporting references, such as the reference title, PMID, DOI, and the relevant sentences where the relationships were identified. Each relationship is supported by one or more references, with each reference corresponding to a single row in S1 Table.*

*Detailed meta-analysis results for each of these genes are provided in Supporting Material S2 Table. The table includes the meta-analysis model used, the number of datasets (studies) involved, log fold change (LFC; column ‘mT’), p-value (column ‘p1’), and the results of multiple linear regression (MLR) analysis. Specifically, the columns ‘Country’, ‘nSample’, and ‘studyAge’ provide the p-values from the MLR analysis, indicating the significance of the influence of country of origin, sample size, and study date on a specific gene.*

*See Supporting Material S3 Table for details of the supporting references, which follow the same format as S1 Table.*

- Please double check the affiliations and the spelling of the authors.

Answer: All author affiliations and spellings have been double-checked and are confirmed to be correct.

- Please also check all the references for being valid and accurate in the journal formatting.

Answer: All references have been thoroughly checked to ensure they are valid, accurate, and formatted according to the journal's requirements.
